# LK or IGF1R: When selectivity hurts

**DOI:** 10.18632/aging.100753

**Published:** 2015-05-30

**Authors:** Igor Puzanov, Alexandra Hess

**Affiliations:** Vanderbilt-Ingram Cancer Center, Nashville, TN, USA

The success of small molecule tyrosine kinase (TKI) inhibitors in cancer treatment and recent studies both in vitro and in vivo sparked interest in targeting the insulin-like growth factor receptor 1 (IGF1R). The development of several antibodies as well as small molecule inhibitors aimed at this receptor continues to be the subject of numerous preclinical studies and clinical trials.

IGF1R, a tyrosine kinase receptor, regulates growth and metabolism, and is also associated with signaling in aging and disease. In cancer, IGF1R plays a role in cancer cell mobility and metastasis. There are three ligands for the cell surface receptor—insulin-like growth factor 1 (IGF-1), IGF-2 and insulin. Reduced activity of IGF-1 together with reduced activity of growth hormone lead to prolonged life span and protection from age-related damage and diseases, including cancer and diabetes, making inhibition of these pathways a promising target for anti-aging therapies [1].

IGF1R shares high homology with the insulin receptor (IR), making the design of selective small molecule inhibitors difficult. Thus, monoclonal antibodies were developed first to achieve high selectivity for IGF1R and no cross-reactivity with the insulin receptor to avoid problems with metabolic inhibition and insulin resistance. However, the results of the first clinical trials were underwhelming, with little objective responses or clinical benefit, except in selected patients whose tumors harbored well defined, but rare gene fusions [2]. This may be attributed to the ability of cancer cells to circumvent the IGF1R signaling via the insulin receptor, and the fact that most patients entering clinical trials have heavily pre-treated tumors with potentially altered expression of IGF1R. In fact, inhibition of IGF1R may lead to increased IGF-1, which results in enhanced insulin receptor signaling that in turn drives tumor growth [3]. In addition, the IGF1R and IR form complex multi-subunit structures that exist as dimers and assemble as combinations of IGF1R and IR subunits (heterodimers) or IGF1R-IGF1R and IR-IR (homo-dimers). The antibodies inhibit only the set of IGF1R-IGF1R homodimers, allowing the signaling to continue via the heterodimer and IR-IR homodimer.

Does this mean that inhibition of both receptors is better? Small molecule TKIs inhibit both IGF1R and IR, including heterodimer and IR-IR homodimer conformations.

We conducted a phase I trial of OSI-906 (linsitinib), an IGF1R TKI, in patients with advanced solid tumors [4]. The drug was well tolerated when administered by once-daily or twice-daily continuous dosing schedule and resulted in decreased phosphorylation of IGF1R and IR in peripheral blood mononuclear cells and increased plasma levels of IGF-1, an indirect measure of IGF1R inhibition. A significant proportion of patients with colorectal cancer experienced stable disease as their best clinical response. Notably, we included a cohort of patients with type 2 diabetes mellitus who had good tolerability of this treatment.

In another phase I study in patients with solid tumors, an intermittent regimen of OSI-906 was associated with antitumor activity in two patients with adrenocortical carcinoma achieving partial responses. However, recently published phase III trial of OSI-906 in patients with adrenocortical carcinoma didn't show improved overall survival compared to placebo; but, good safety profile and long-lasting partial responses were observed in three patients, indicating some therapeutic potential in this patient group [5].

Poor response in these and other trials may be attributed to low impact of IGF1R on tumor proliferation and the ability of the tumor to circumvent this pathway. Inhibition of activated IGFR1R in cancer cells may not necessarily curb the proliferation but suppress metastasis; however, this may not be identified in typical phase II trials designed to look at tumor response. Careful planning of the sequence of treatments as well as primary and secondary outcomes is critical in the design of potentially active combinations of IGF1R inhibitors with other therapies. While IGF1R inhibitors may work alone, albeit rarely, combination therapy holds promise for anti-IGF1R agents. Effective therapy via combined inhibition of IGF1R and other tyrosine kinases may work in some cancers. Tyrosine kinase inhibitors are successfully used in many cancers, but ultimately resistance develops, in some cases via signaling through IGF1R pathway, like with the epidermal growth factor receptor (EGFR) and anaplastic lymphoma tyrosine kinase (ALK). Based on a patient with ALK fusion–positive lung cancer who had an exceptional response to an IGF1R-specific antibody, Lovly et al showed that chronically inhibited ALK enhanced IGF1R signaling and that ALK and IGF1R inhibitors, including OSI-906, together have increased antiproliferative effects [6].

How can we prevent failure of these combinations despite encouraging pre-clinical data? The key is to develop better biomarkers to identify patients who may benefit. Since levels of IGF1R expression are a weak predictor of benefit as they fail to show receptor conformation, methods such as the proximity ligation assays need to be developed. Given the role EGFR and IGF1R in obesity and aging, and correlation of some cancers with obesity and diabetes, these targets are certainly worth pursuing in combination [7].
